# CRIF1 gene therapy ameliorates inflammatory bowel disease by suppressing TH17 cells and fibrosis through mitochondrial function regulation

**DOI:** 10.3389/fimmu.2025.1618012

**Published:** 2025-07-31

**Authors:** Jin-Sil Park, Hye Yeon Kang, Ha Yeon Jeong, SeungCheon Yang, JeongWon Choi, Sang Hee Cho, Sol Kim, Bo-In Lee, Mi-La Cho

**Affiliations:** ^1^ Lab of Translational ImmunoMedicine, Catholic Research Institute of Medical Science, College of Medicine, The Catholic University of Korea, Seoul, Republic of Korea; ^2^ Department of Pathology, College of Medicine, The Catholic University of Korea, Seoul, Republic of Korea; ^3^ The Rheumatism Research Center, Catholic Research Institute of Medical Science, College of Medicine, The Catholic University of Korea, Seoul, Republic of Korea; ^4^ Department of Medical Sciences, Graduate School of The Catholic University of Korea, Seoul, Republic of Korea; ^5^ Divisions of Gastroenterology and Department of Internal Medicine, Seoul St. Mary’s Hospital, College of Medicine, The Catholic University of Korea, Seoul, Republic of Korea

**Keywords:** inflammatory bowel disease, CRIF1, mitochondria, inflammatory cytokine, fibrosis

## Abstract

**Background:**

CR6-interacting factor 1 (CRIF1) is a nuclear transcriptional regulator and a mitochondrial inner membrane protein. Although serious modifications of the tissue architecture of the small intestine have been reported in CRIF1-deficient mice, how this may affect the development of inflammatory bowel disease (IBD) remains unclear. We investigated the effects of CRIF1 on mice with colitis.

**Methods:**

In DSS-induced colitis mice administered p3XFLAG-CMV-10-CRIF1, clinical symptoms were evaluated. Mitochondrial morphology in the intestinal tissues of colitis mice and UC patients was observed by electron microscopy. Level of CRIF1 in the splenic mitochondria of colitis mice or human PBMCs were investigated by western blot or real-time PCR, and the amount of IL-17 in the supernatant of healthy PBMCs co-cultured with CRIF1-overexpressing mitochondria was investigated by ELISA.

**Results:**

Overexpression of CRIF1 attenuated the severity of colitis, alleviated weight loss, and intestinal shortening. Moreover, overexpression of CRIF1 significantly reduced the levels of proinflammatory and necroptosis-related factors in colon and inhibited intestinal fibrosis. The intestines of these mice showed a reduced level of CRIF1 and altered mitochondrial morphology. Transplantation of CRIF1-overexpressed mitochondria into mice with colitis alleviated disease severity. Patients with ulcerative colitis exhibited decreased CRIF1 levels with dysfunctional mitochondria in inflamed colonic tissue. CRIF1-overexpressing mitochondria inhibited IL-17 production in PBMCs from healthy control.

**Conclusion:**

Our findings demonstrate that CRIF1 alleviates IBD by suppressing inflammation and fibrosis by improving mitochondrial function. Improving mitochondrial function through CRIF1 may be a potential therapeutic strategy for IBD.

## 1 Introduction

Inflammatory bowel disease (IBD) is a chronic and relapsing inflammatory disease of the gastrointestinal tract caused by multiple genetic and environmental factors and an abnormal immune response (1, 2). IBD is divided into two major clinical subtypes: Crohn’s disease (CD), which results in transmural chronic ulceration of any portion of the gastrointestinal tract, and ulcerative colitis (UC), which comprises mucosal inflammation of the large intestine only (3). The global incidence of IBD has markedly increased over the 21st century but varies widely by region, ranging from 0.1 to 58 cases per 100,000 person-years to 0.9 to 505 cases per 100,000 population; the highest age-standardized prevalence rate in 2017 was reported in the United States, followed by the UK (4, 5). Intestinal fibrosis, characterized by increased deposition of extracellular matrix components, is common in IBD, ultimately leading to dysfunctional wound healing and colonic wall thickening (6, 7). However, the etiology of IBD remains uncertain. There is evidence that intestinal mucosal dysfunction and abnormal intestinal epithelial cells and immune cells are important factors (8, 9). Dysregulated lamina propria cells including T cells, B cells, macrophages, and neutrophils produce large amounts of proinflammatory cytokines, such as tumor necrosis factor α (TNF-α), interleukin (IL)-1β, and IL-23/Th17-related cytokines, in local tissues (10, 11). This cytokine production promotes excess production of extracellular matrix components, amplifies the inflammation cascade, and thus facilitates the development of intestinal fibrosis and intestinal distortion (12).

There is increasing evidence that mitochondrial injury and dysfunction (also known as mitochondriopathy) are involved in IBD. In one study of 408 active UC patients, the expression of mitochondrial-encoded and nuclear-encoded mitochondrial genes was significantly suppressed (13); in another study, severe mitochondrial damage was observed in Paneth cells, goblet cells, and enterocytes during active CD inflammation (14). The level of circulating mitochondrial DNA in plasma is significantly increased in UC and CD; it is positively correlated with disease activity and severity (15). The mitochondrial unfolded protein response is implicated in the pathophysiology of epithelial cells in IBD patients (16).

CR6-interacting factor 1 (CRIF1) functions as a transcriptional factor for cell cycle and growth and as a mitochondrial protein associated with synthesis and insertion of mitochondrial oxidative phosphorylation (OXPHOS) polypeptides into the mitochondrial membrane (17, 18). It acts as a transcriptional coactivator of E74-like factor 3 in the differentiation of intestinal epithelial cells (19). Mice without CRIF1 in the intestinal epithelium undergo abnormal cell differentiation, which can lead to severe abnormal morphogenesis and defective terminal differentiation of enterocytes (19). However, information on the role of CRIF1 in IBD remains insufficient.

To address this, we investigated changes in CRIF1 expression during disease development in mice with colitis and evaluated the therapeutic efficacy of CRIF1 overexpression and transplantation of CRIF1-overexpressing mitochondria. We also evaluated the inflammation-modulating outcome of coculture of CRIF1-overexpressing mitochondria with PBMCs from human.

## 2 Materials and methods

### 2.1 Mice

Eight-week-old male C57BL/6 mice were purchased from Orient Bio Inc. (Seongnam, Korea). The animals were maintained under specific-pathogen-free conditions at the Institute of Medical Science of Catholic University of Korea and were housed under controlled temperature (21–22°C) and light (12/12 h light/dark cycle) conditions with standard mouse chow and water. All experimental procedures were approved by the Department of Laboratory Animals Institutional Animal Care and Use Committee of the School of Medicine, Catholic University of Korea, and conformed with all United States National Institutes of Health guidelines (Permit number: CUMC 2016-0247-03; CUMC 2017–0126–02; CUMC 2021-0050-01). For colitis induction, C57BL/6 mice were administered 3% DSS (MP Biomedicals, Santa Ana, CA, USA, #02160110) in their drinking water for 5 days; they were given regular drinking water beginning on day 5. After 5 days of DSS administration, the mice were weighed and assigned to control and treatment groups based on their average body weight. For CRIF1 overexpression, p3XFLAG-CMV-10-CRIF1 or mock vector was intravenously injected 1 day before and on day 7 from the start of 3% DSS treatment (20). For the mitochondria-transplant experiment, mitochondria were isolated from C2C12 cells and then intraperitoneally injected into mice (10 μg in 200 μL saline) 1 day before and on days 2, 4, 6, 9, 12, and 14 from the start of 3% DSS treatment (n = 5/group). The sample size was determined as the minimum number required for statistical significance. During the experimental period, weight changes and the disease activity index (DAI) were evaluated to assess disease severity. DAI was evaluated based on weight loss, stool consistency, and visible gross bleeding, in accordance with a previously reported method (21).

### 2.2 Histopathological analysis

Colon tissues were fixed with 10% (v/v) neutral-buffered formalin (Sigma-Aldrich). Sections (5 μm) were stained with hematoxylin and eosin and examined via photomicroscopy (Olympus, Tokyo, Japan) (magnification: 40×), using a scoring system based on four parameters (22). Epithelial loss was scored as follows: 0, no loss; 1, 0–5% loss; 2, 5–10% loss; and 3, >10% loss. Crypt damage was scored as follows: 0, no damage; 1, 0–10% loss of crypts; 2, 10–20% loss of crypts; 3, >20% loss of crypts. Goblet cell depletion was scored as follows: 0, none; 1, mild; 2, moderate; and 3, severe. Inflammatory cell infiltration was scored as follows: 0, none; 1, mild; 2, moderate; and 3, severe. Scores were determined by two observers in a blinded manner, and the total score was the sum of each individual score. Masson’s trichrome staining was performed using a kit (Polysciences, Warrington, PA, USA, #25088–100) to observe changes in collagen deposition in the colon. Stained colon tissues were examined via photomicroscopy (Olympus; magnification: 200×).

### 2.3 Immunohistochemistry

Colon sections were immunohistochemically analyzed using the Envision Detection kit (DAKO, Glostrup, Denmark, #5007). The sections were incubated with primary antibodies against TNF-α (1:50, Abcam, Cambridge, UK, #ab6671), IL-1β (1:100, Abcam, #ab9722), IL-17 (1:200, Abcam, #ab79056), IL-6 (1:200, Abcam, #ab7737), IL-8 (1:100, Novus, #NBP2-33819), CRIF1 (1:250, Thermo Fisher, #BS-6008R; Abcam, #ab244530), TNFR1 (1:1000, Santa Cruz, # SC-7895), RIPK1 (1:400, Invitrogen, #PA5-20811), RIPK3 (1:200, Thermo Fisher, #PA5-19956), phosphorylated MLKL (1:50, Thermo Fisher, #PA5-105677), α-smooth muscle actin (α-SMA) (1:2500, Abcam, #ab7817) and type I collagen (Col-I) (1:100, Abcam, #ab6308) for 2 h at room temperature. The sections were then incubated with a horseradish peroxidase-conjugated secondary antibody for 30 min. The final products were developed using the chromogen diaminobenzidine. Immunostained sections were examined via photomicroscopy (Olympus). The numbers of positive cells were counted in high-power digital images (magnification: 400×) using Adobe Photoshop software (Adobe, San Jose, CA, USA). Positive cells were visually enumerated by three individuals, and the mean values were calculated.

### 2.4 Electron microscopy

Colonic tissue from mice with DSS-induced colitis or UC patients was fixed with 4% paraformaldehyde and 2.5% glutaraldehyde in 0.1 M phosphate buffer overnight at 4°C. The tissue was washed in 0.1 M phosphate buffer, postfixed with 1% osmium tetroxide for 1 h at 4°C, dehydrated in graded ethyl alcohol solutions, exchanged in acetone, and embedded in Epon 812. Ultrathin sections (70–80 nm) were obtained with an ultramicrotome (Leica Ultracut; Leica, Vienna, Austria) and stained with uranyl acetate and lead citrate. Images were acquired at 60 kV using a transmission electron microscope (TEM, JEM 1010; JEOL, Tokyo, Japan).

### 2.5 Transfection

C2C12 cells cells (Korean cell line bank, Seoul, Korea), which are myoblasts from mouse muscle, were transfected with p3XFLAG-CMV-10-CRIF1 or mock vector using Lipofectamine, in accordance with the manufacturer’s recommendation. After 3 days, the cells were harvested and the mitochondrial fraction was isolated in accordance with the manufacturer’s protocol. CCD-18Co cells (Korean cell line bank), a non-malignant fibroblast cell line isolated from normal human colon tissue, were transfected with pCMV6-Myc-DDK hCRIF1 vector or mock vector using lipofectamine. For immunoblot assays, 3 days after transfection, CCD-18Co cells were starved for 24 h with serum-free DMEM and stimulated with 10 ng/mL TGF-β (Peprotech, #100-21C-10) for 24 h to obtain protein from the cells.

### 2.6 Isolation of mitochondria from C2C12 cells, CCD-18Co cells, or murine splenocytes

Mitochondria were isolated from C2C12 cells, CCD-18Co cells, or splenocytes from mice with DSS-induced colitis using the Mitochondrial Isolation Kit for Cultured Cells (Thermo, Waltham, MA, USA, #89874) in accordance with the manufacturer’s protocol. Briefly, cells were harvested and incubated on ice for 2 min with 400 µL reagent A, vortexing every minute; 5 µL reagent B were then added and the samples were incubated on ice for 5 min. Next, 400 µL reagent C were added, and the samples were centrifuged at 700 × g for 10 min at 4°C. The supernatants were transferred to new tubes, which were centrifuged at 12,000 × g for 15 min at 4°C. Finally, 400 µL reagent C were added to the mitochondrial pellet, and the sample was centrifuged at 12,000 × g for 5 min at 4°C to obtain the mitochondrial fraction.

### 2.7 Isolation of human peripheral blood mononuclear cells and cell stimulation

Human blood was obtained from healthy controls and patients with IBD. All patients gave informed written consent. This study was approved by the Institutional Review Board of Seoul St. Mary’s Hospital (XC18TEDI0027) and was performed in accordance with the Helsinki II Declaration. Clinical baseline characteristics are summarized in **Supplementary Table 1**. Peripheral blood mononuclear cells (PBMCs) were separated from the buffy coat using Ficoll–Hypaque (Pharmacia Biotech, Piscataway, NJ, USA). The cells were cocultured with mitochondria from CCD-18Co cells that had been transfected with pCMV6-Myc-DDK hCRIF1 vector or mock vector in the presence of anti-CD3 antibody (2 μg/mL, BD Pharmingen, #553057). After 3 days, the supernatant was harvested.

### 2.8 Enzyme-linked immunosorbent assay

The IL-17 levels in culture supernatants were determined using a sandwich enzyme-linked immunosorbent assay (ELISA) with 96-well plates coated with anti-mouse IL-17 capture Ab (#DY421; R&D Systems) and incubated overnight at 4˚C temperature. After the overnight incubation, the plates were blocked with 200 μL phosphate-buffered saline containing 1% bovine serum albumin and 0.05% Tween 20 for 2 h at room temperature. Mouse IL-17 standard was diluted twofold from 1000 to 15.6 pg/mL. Culture supernatant or standards in reagent diluent were added to the plates and incubated at room temperature for 2h. Subsequently, the plates were washed, 50 μL of detection Ab, diluted in reagent diluent, was added to each well, and incubated for 2 h at room temperature. The plates were washed and then 50 μL of working dilution of Streptavidin-HRP B was added to each well, and incubated for 20 min at room temperature. The plates were washed and then 50 μL of substrate solution was added to each well, and incubated for 20 min at room temperature. 50 μL of stop solution was added to each well and absorbance at 450 nm was measured on an ELISA microplate reader (Molecular Devices).

### 2.9 Real-time polymerase chain reaction

Total RNA was extracted using TRI Reagent (Molecular Research Center), and cDNA was synthesized with Dyne first-strand cDNA synthesis kit (Dyne Bio) according to the manufacturer’s protocol. Polymerase chain reaction amplification and analysis were performed using a LightCycler 2.0 instrument (Roche Diagnostics, Indianapolis, IN, USA) and the accompanying software (version 2.3). TaqMan™ Fast Advanced Master Mix for qPCR (Applied Biosystems, #4444557) and site-specific primers and probes (CRIF1: Hs00369339_m1 and GAPDH: Hs02786624_g1; Applied Biosystems) were used for quantitative polymerase chain reaction.

### 2.10 Intracellular staining and flow cytometry

For surface marker staining, single-cell suspensions were washed with FACS buffer (PBS with 2% FBS) and incubated with fluorochrome-labeled antibodies for 30 min at 4°C. For intracellular staining, single-cell suspensions were cultured with 25 ng/mL PMA (Sigma-Aldrich, p8139) and 250 ng/mL ionomycin (Sigma-Aldrich, I0634) with the addition of GolgiStop (BD Biosciences, #554715) for 4 h. After surface staining, cells were fixed and permeabilized with Cytofix/Cytoperm, in accordance with the manufacturer’s instructions (BD Biosciences, #554715). For intracellular Foxp3, a Foxp3 Staining Buffer kit was used (eBiosciences, #00-5523-00) after surface staining. After washing with Perm/Wash buffer, antibodies for intracellular staining were added for 30 min at 4°C. The following anti-mouse antibodies were used for FACS: Alexa Fluor^®^ 700 anti-CD4 (RM4–5) and PE-Cy7 anti-CD19 (1D3) were from BD Biosciences; PerCP-Cy5.5 anti-CD4 (RM4–5), eFluor 450 anti-CD4 (RM4–5), FITC anti-CD5 (53–7.3), PE anti-CD1d (1B1), PE anti-Foxp3 (FJK-16s), PE anti-IFN-γ (XMG1.2), PE anti-IL-4 (11B11), PE anti-IL-17 (eBio17B7), and APC anti-IL-10 (JES5–16E3) were from Invitrogen; and APC anti-CD25 (PC61) was from BioLegend. Stained cells were analyzed on a FACSCalibur (BD Biosciences), CytoFLEX (Beckman Coulter), or LSRII (BD Biosciences) device. Events were recorded and analyzed using FlowJo software (Tree Star).

### 2.11 Immunoblot analysis

Cells were lysed in Halt protein lysis buffer containing Halt phosphatase inhibitor (Thermo Fisher, #78440). Lysates were centrifuged at 14,000 × *g* for 15 min at 4°C. Protein concentrations were determined using the Bradford protein assay (Bio-Rad). Proteins were electrophoretically separated via sodium dodecyl sulfate–polyacrylamide gel electrophoresis (SDS-PAGE) and transferred to Hybond ECL membranes (Cytiva, #10600001). Membranes were incubated with antibodies against Col1a1 (Thermo Fisher, #PA5-89281), fibronectin (Abcam, #ab2413), and β-actin (Santa Cruz, #SC-47778). Hybridized bands were detected using an ECL detection kit (Pierce) and Hyperfilm (Agfa). Western blotting analysis was performed using the SNAP i.d. Protein Detection System (Millipore).

### 2.12 Statistical analysis

All statistical analyses were performed using GraphPad Prism (v.4 for Windows; GraphPad Software, Inc., La Jolla, CA, USA). Normally distributed continuous data were analyzed using the parametric Student’s *t*-test. Differences in mean values among groups were subjected to analysis of variance. Data are presented as means ± standard deviations (SDs). Values of *P* < 0.05 (two-tailed) were considered statistically significant.

## 3 Results

### 3.1 CRIF1 overexpression alleviates clinical symptoms of DSS-induced colitis

**Figure 1:**
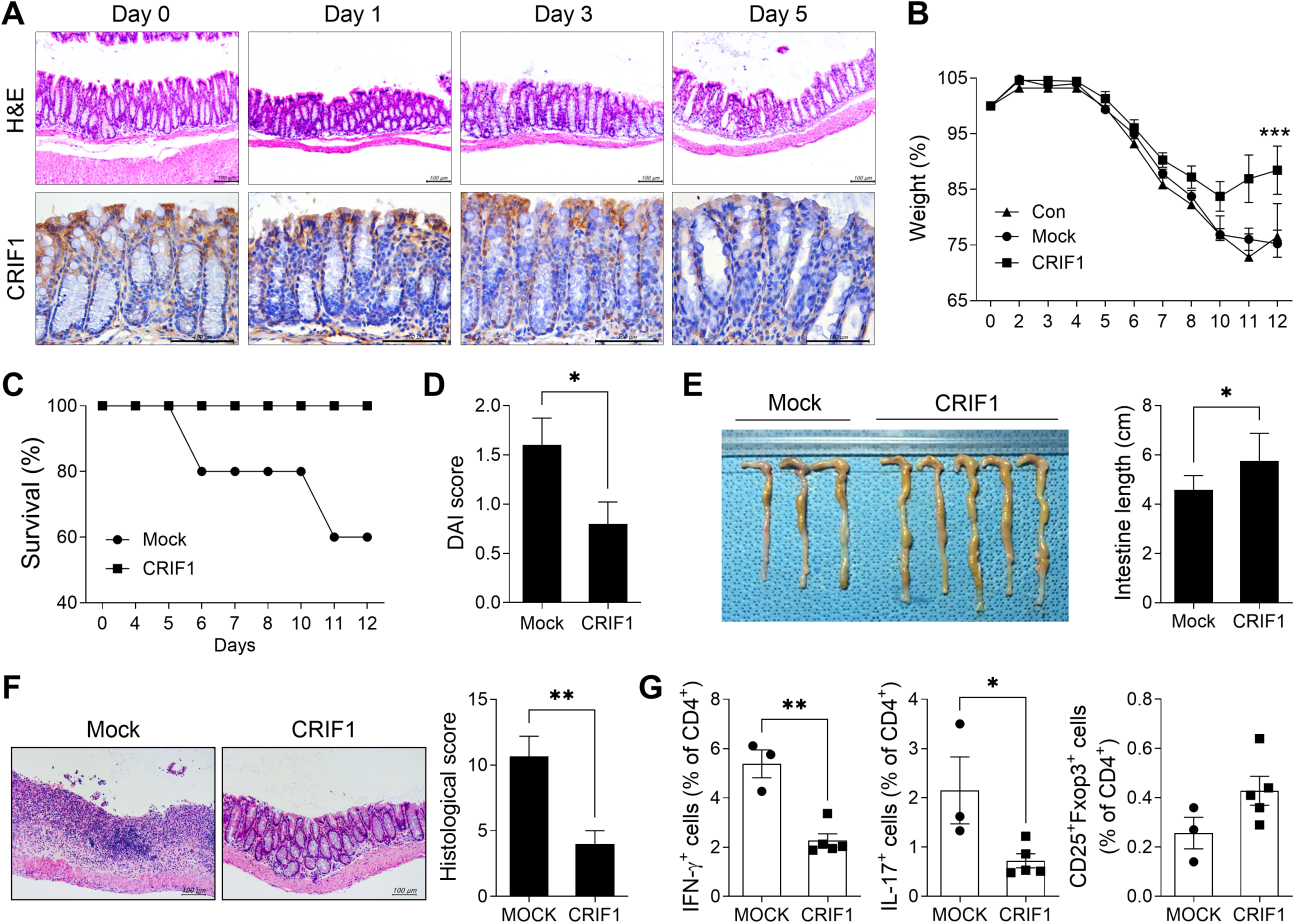
CRIF1 alleviates the development of DSS-induced colitis. **(A)** C57BL/6 mice were orally treated with 3% DSS in distilled water; colons were obtained on days 0, 1, 3, and 5. Colon tissue sections were subjected to hematoxylin and eosin staining (upper panel) and immunohistochemistry using antibodies against CRIF1 (lower panel). Representative images are shown. Original magnification: 200×. Scale bar: 100 µm. **(B-G)** Acute colitis was induced in C57BL/6 mice through oral administration of 3% DSS in distilled water for 5 days, followed by regular drinking water for 7 days. p3XFLAG-CMV-10-CRIF1 (n = 5) or mock (n = 3) vector was intravenously injected 1 day before and on day 7 from the start of 3% DSS treatment. **(B)** Changes in body weight were measured, and the percentage change in body weight was calculated based on the initial weight (at day 0). **(C)** Survival rate during the disease process. **(D)** Disease activity index **(DAI)** score on day 12 after treatment. **(E)** On day 12 after treatment, changes in colon length were measured. **(F)** On day 12, colon tissue sections were stained with hematoxylin and eosin. Representative images are shown (original magnification: 200×, scale bar: 100 µm). The graph shows the mean histological scores. **(G)** The frequencies of IFN-γ+CD4+, IL-17+CD4+, and CD25+Foxp3+CD4+ cells in peripheral blood of mice were analyzed via flow cytometry. Values are presented as means ± SDs. *P < 0.05, **P < 0.01, and ***P < 0.001. Data are representative of two independent experiments.

To investigate changes in the level of CRIF1 in the colon during colitis development, the colon of each mouse was obtained on days 1, 3, and 5 after treatment and the expression of CRIF1 was analyzed via immunohistochemistry (**Figure 1A**). H&E staining showed that as the disease progressed, the destruction of crypts in the colon, the loss of the epithelial barrier, and the infiltration of immune cells intensified. Intriguingly, the level of CRIF1 in the colon also decreased; a negative correlation was observed between CRIF1 expression and disease severity. To investigate the effect of CRIF1 overexpression on the development of IBD, p3XFLAG-CMV-10-CRIF1 was intravenously injected 1 day before and on day 7 from the start of 3% DSS treatment, and the efficacy of CRIF1 was evaluated. Overexpression of CRIF1 alleviated weight loss and improved the survival rate compared with controls (**Supplementary Figure S1**; **Figures 1B, C**). Overexpression of CRIF1 significantly lowered the disease activity index (DAI) score and restored colon length on day 12 after treatment (**Figures 1D, E**). Additionally, CRIF1 overexpression prevented the infiltration of inflammatory cells into intestinal tissue and the development of a damaged colon with crypt loss and ulceration (**Figure 1F**). To investigate the effect of CRIF1 overexpression on the regulation of the T cell phenotype, the frequencies of Th1, Th17, and Treg cells in peripheral blood were analyzed via flow cytometry. Overexpression significantly lowered the frequencies of Th1 and Th17 cells and tended to increase the frequency of Treg cells in the peripheral blood of treated mice (**Figure 1G**). These results indicate that CRIF1 has therapeutic efficacy in mice with DSS-induced colitis.

### 3.2 CRIF1 suppresses the level of inflammatory mediators in the colon in DSS-induced colitis

**Figure 2:**
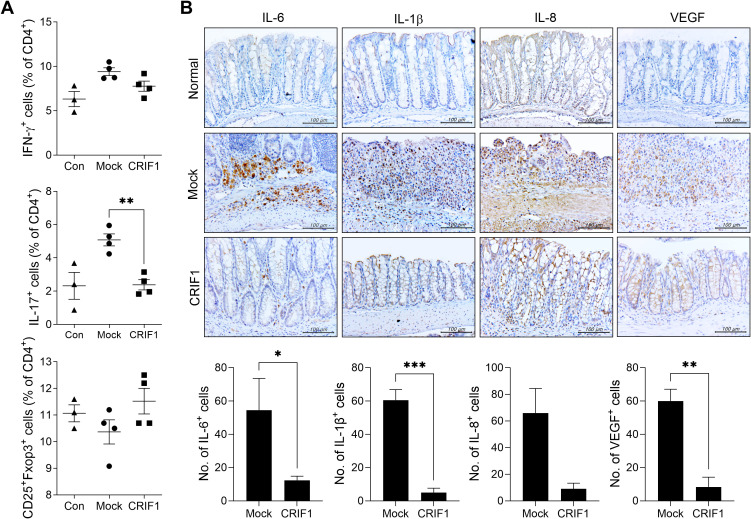
CRIF1 reduces colonic inflammation in mice with DSS-induced colitis. **(A)** Acute colitis was induced in C57BL/6 mice through oral administration of 3% DSS in distilled water for 5 days, followed by regular drinking water for 8 days. p3XFLAG-CMV-10-CRIF1 (n = 4) or mock (n = 4) vector was intravenously injected 1 day before and on day 7 from the start of DSS treatment. On day 13 after DSS administration, the frequencies of IFN-γ+CD4+, IL-17+CD4+, and CD25+Foxp3+CD4+ cells in *ex vivo* spleens of mice were analyzed via flow cytometry (normal mice, n = 3). **(B)** Colitis was induced in C57BL/6 mice through oral administration of 3% DSS in distilled water for 5 days, followed by regular drinking water for 7 days. p3XFLAG-CMV-10-CRIF1 or mock vector was intravenously injected 1 day before and on day 7 from the start of treatment. On day 12 after treatment, colon sections were stained with antibodies against IL-6, IL-1β, IL-8, and VEGF. Representative images are shown (original magnification: 400×, Scale bar: 100 µm). Graphs show the numbers of antibody-positive cells. Values are presented as means ± SDs. *P < 0.05, **P < 0.01, and ***P < 0.001. Data are representative of two independent experiments.

In mice with colitis, overexpression of CRIF1 significantly lowered the frequency of Th17 cells in the spleen. Furthermore, although the differences were not statistically significant, Th1 cells and Treg cells in spleen showed tendencies to decrease and increase, respectively (**Figure 2A**). To investigate the effect of CRIF1 on intestinal inflammation, the levels of inflammatory mediators were examined via immunohistochemistry in colon sections from CRIF1-overexpressing mice with colitis. CRIF1 overexpression effectively reduced the infiltration of cells expressing IL-6, IL-1β, IL-8, or VEGF within the intestinal tissue (**Figure 2B**), demonstrating that CRIF1 can mitigate colonic inflammation.

### 3.3 CRIF1 reduces the levels of mediators related to inflammatory cell death in the colon of mice with colitis

**Figure 3:**
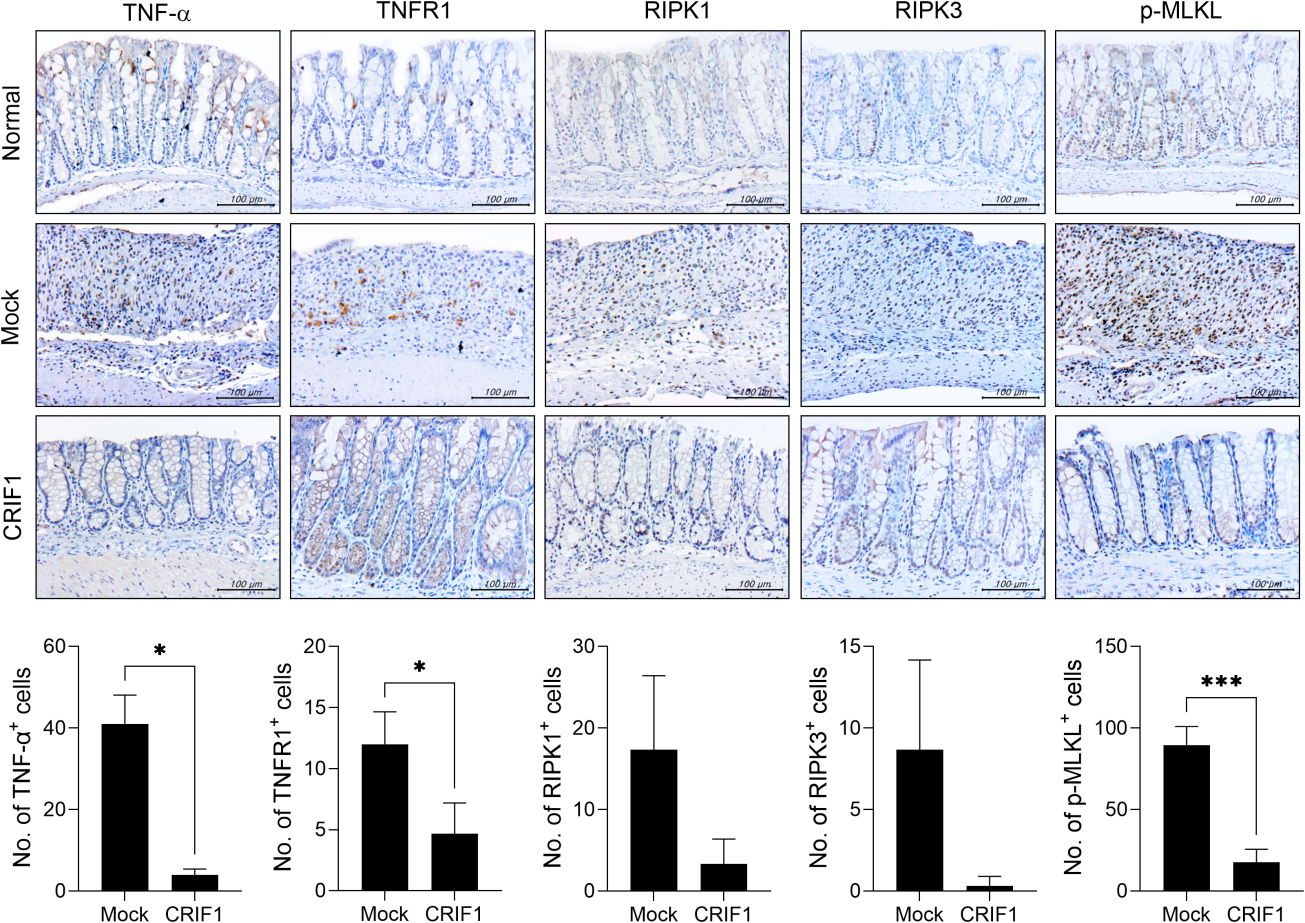
CRIF1 reduces the levels of mediators associated with necroptosis. Colitis was induced in C57BL/6 mice through oral administration of 3% DSS in distilled water for 5 days, followed by regular drinking water for 7 days. p3XFLAG-CMV-10-CRIF1 or mock vector was intravenously injected 1 day before and on day 7 from the start of treatment. On day 12 after treatment, colon sections were stained with antibodies against TNF-α, TNFR1, RIPK1, RIPK3, and phosphorylated MLKL. Representative images are shown (original magnification: 400×, scale bar: 100 µm). Graphs show the numbers of antibody-positive cells. Values are presented as means ± SDs. *P < 0.05, and ***P < 0.001. Data are representative of two independent experiments.

Immune ligands such as TNF-α lead to the activation of RIPK3, which phosphorylates MLKL and mediates necroptosis (23, 24). Necroptosis increases in mice with colitis, and the suppression of necroptosis via RIPK3 inhibition alleviates the disease (25). We previously reported that CRIF1 deficiency increases necroptic cells (27). To determine whether CRIF1 overexpression reduces necroptosis in the colon of these mice, we examined the levels of necroptosis-related mediators in colon sections. Indeed, CRIF1 overexpression reduced the numbers of cells expressing TNF-α, TNFR1, RIPK1, RIPK3, and phosphorylated MLKL in our experimental mice (**Figure 3**), suggesting that CRIF1 overexpression can downregulate intestinal inflammatory cell death in colitis.

### 3.4 CRIF1 mitigates the development of fibrosis in mice with colitis

**Figure 4:**
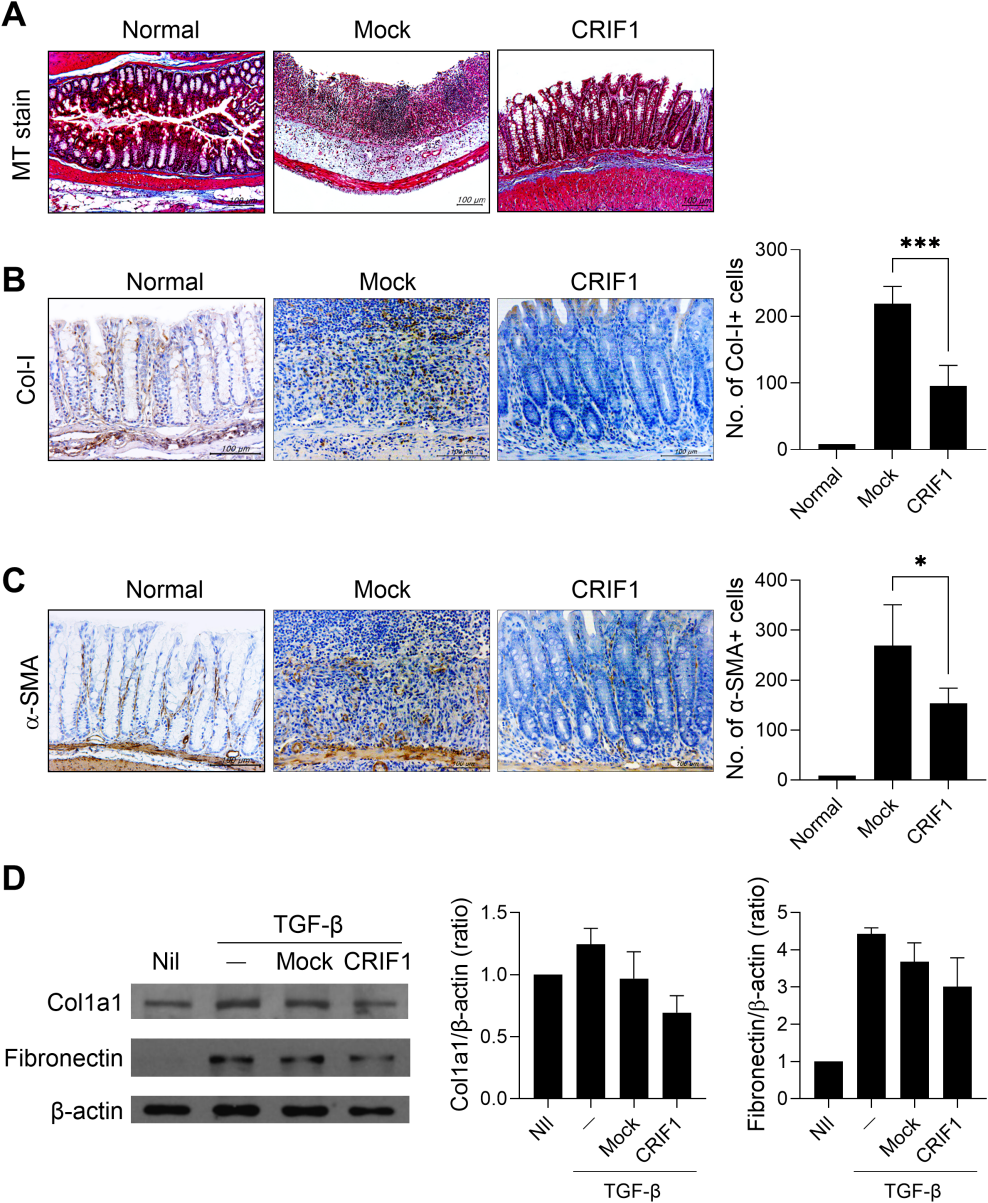
CRIF1 mitigates the development of fibrosis in colonic mucosa. Colitis was induced in C57BL/6 mice through oral administration of 3% DSS in distilled water for 5 days, followed by regular drinking water for 7 days. p3XFLAG-CMV-10-CRIF1 or mock vector was intravenously injected 1 day before and on day 7 from the start of treatment. **(A)** On day 12 after treatment, colon sections were stained with Masson’s trichrome. Representative images are shown (original magnification: 200×, scale bar: 100 µm). **(B, C)** Colon sections were immunohistochemically stained for collagen-I (Col-I) and α-smooth muscle actin (α-SMA). Representative images are shown (original magnification: 400×, scale bar: 100 µm). Graphs show the numbers of antibody-positive cells. **(D)** CCD-18Co Cells were transfected with pCMV6-Myc-DDK hCRIF1 vector or mock vector for 3 days, then starved for 24 h with serum-free DMEM and stimulated with 10 ng/mL TGF-β for 24 h. The levels of Col1a1 and fibronectin were assessed by immunoblotting. Values are presented as means ± SDs. *P < 0.05, and ***P < 0.001. Data are representative of two independent experiments.

To investigate the antifibrotic effect of CRIF1 in colitis-associated fibrosis, colon sections were stained with Masson’s trichrome. In mice with colitis, intestinal structure was lost due to dense extracellular matrix deposition and infiltration of inflammatory cells; collagen deposition also increased. However, in CRIF1-overexpressing mice, the structure remained relatively intact and collagen deposition was improved (**Figure 4A**). Notably, the number of Col-I+ cells decreased in CRIF1-overexpressing mice (**Figure 4B**). To determine whether CRIF1 inhibits the differentiation of fibroblasts into myofibroblasts, an important step in the fibrogenic process of chronic colitis, intestinal tissue was stained for α-SMA, a marker of activated myofibroblasts. The number of α-SMA+ cells decreased in the colonic mucosa of diseased CRIF1-overexpressing mice (**Figure 4C**). Subsequently, to investigate the effect of CRIF1 overexpression on fibrosis, we transfected CCD-18Co cells, a human fibroblast cell line, with a pCMV6-Myc-DDK hCRIF1 vector and evaluated the effect on fibrosis activity regulation via TGF-β stimulation. CRIF1 overexpression suppressed the levels of the fibrosis-related markers Col1a1 and fibronectin, which were induced by TGF-beta stimulation (**Figure 4D**). These results show that CRIF1 mitigates the development of fibrosis in colonic mucosa.

### 3.5 Mice with DSS-induced colitis exhibit altered mitochondria and decreased levels of mitochondrial CRIF1

**Figure 5:**
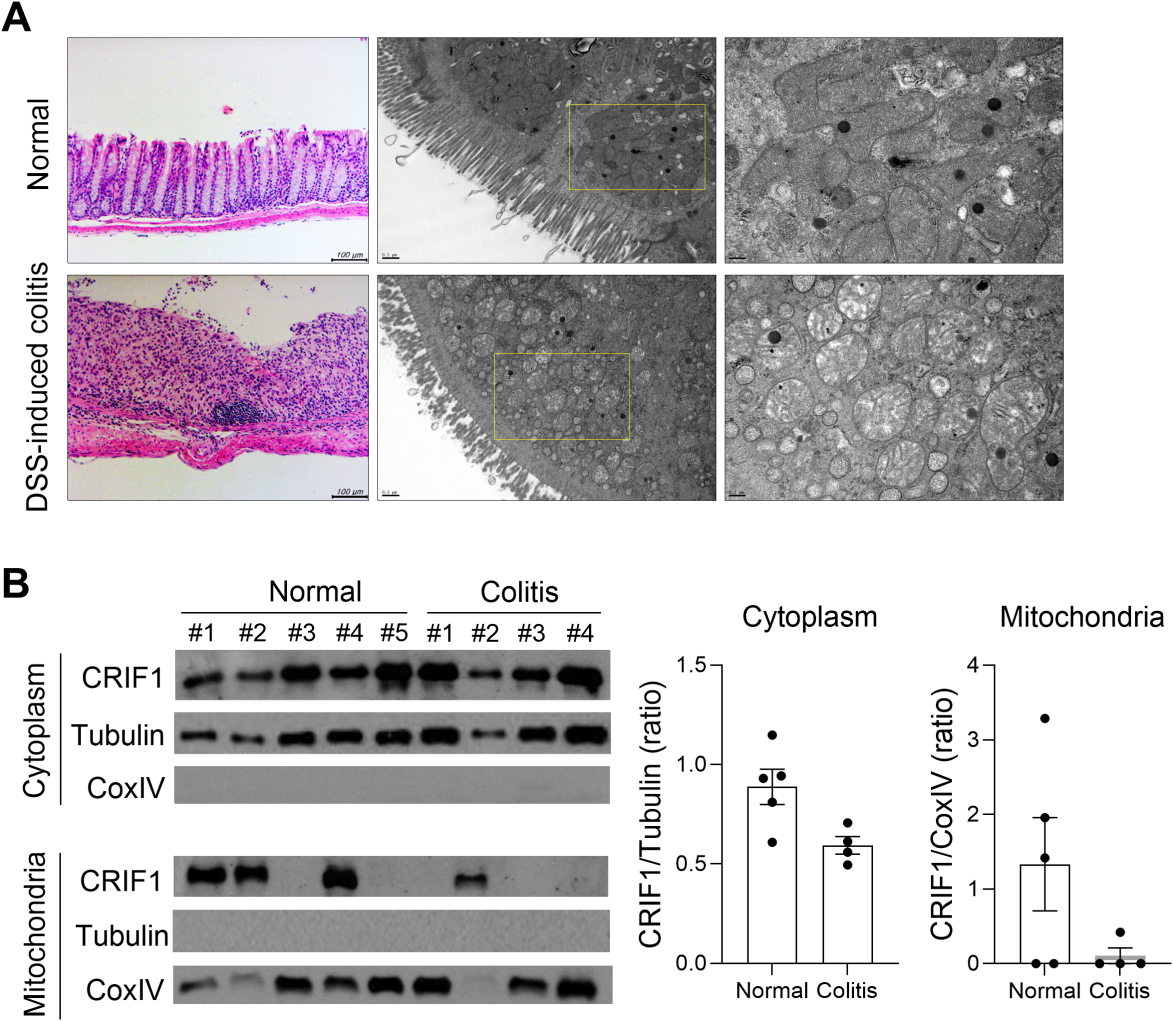
Mice with DSS-induced colitis exhibit mitochondrial modifications and reduced expression of CRIF1. **(A-B)** Acute colitis was induced in C57BL/6 mice through oral administration of 3% DSS in distilled water for 5 days, followed by regular drinking water for 7 days. **(A)** On day 12 after DSS administration, structural changes in mitochondria were examined in colon tissue sections via transmission electron microscopy. Representative images are shown. **(B)** Cytosol and mitochondrial fractions were obtained from the spleen, and the level of CRIF1 was examined via Western blotting. Tubulin and COX IV were used as validation controls for cytosolic and mitochondrial fractions, respectively. The graph shows the ratio of the relative density of the CRIF1 protein normalized to each validation control.

To investigate changes in mitochondria in the colon of IBD, colitis was induced in normal mice by administering 3% DSS for 5 days; the large intestine was observed via TEM on day 12. The treatment led to significant damage to the large intestine, including swollen mitochondria with enlarged and broken cristae and a dilated endoplasmic reticulum (**Figure 5A**). To investigate changes in CRIF1 levels within the mitochondria of treated mice, protein levels were measured from the cytoplasm and mitochondrial fractions of the spleens of normal mice and those with colitis (**Figure 5B**). Compared with controls, CRIF1 in treated mice tended to be lower in the cytoplasm, but no CRIF1 was evident in the mitochondrial fraction. These results show that CRIF1 levels decreased along with mitochondrial impairment in mice with colitis.

### 3.6 Transplantation of CRIF1-overexpressing mitochondria alleviates DSS-induced colitis

**Figure 6:**
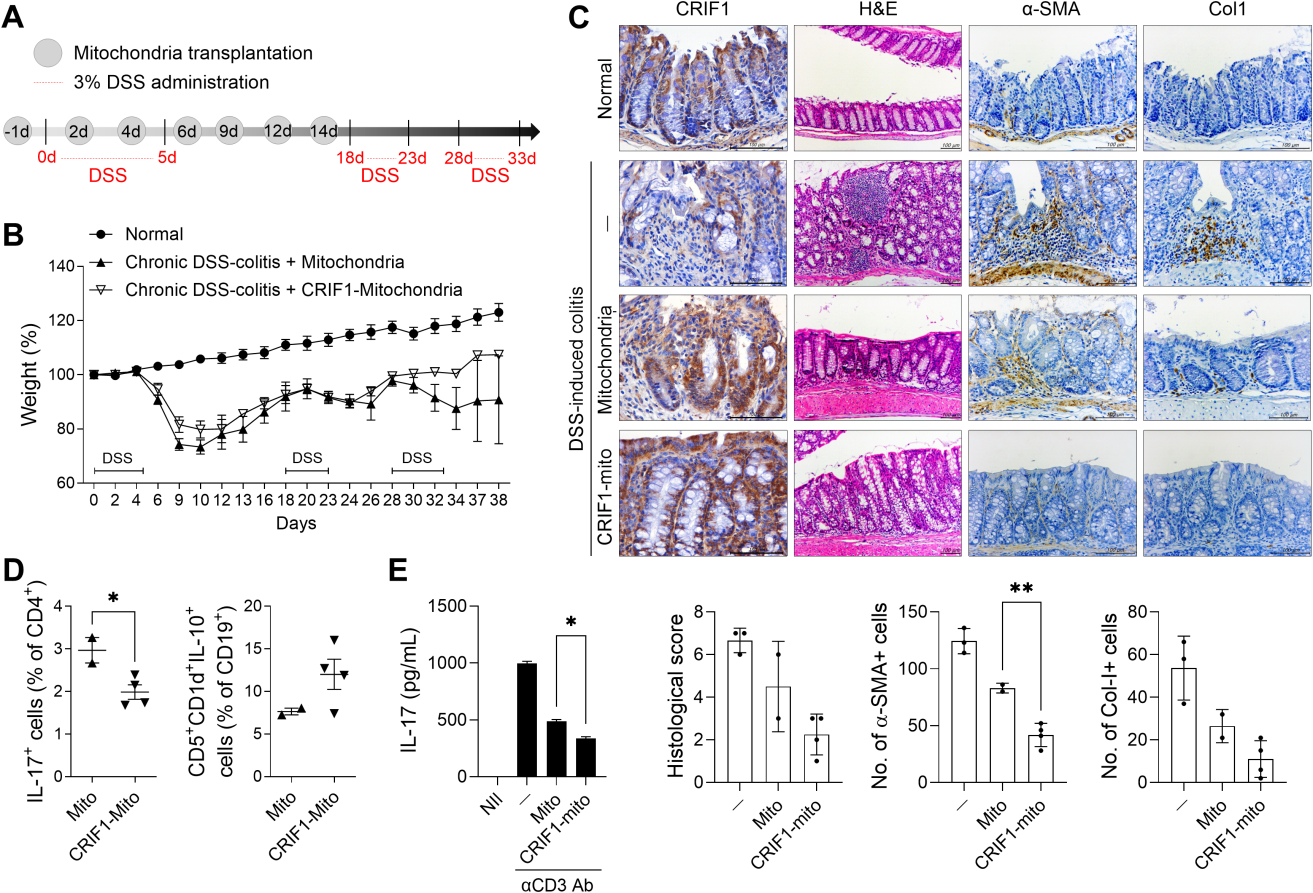
Transplantation of CRIF1-overexpressing mitochondria alleviates DSS-induced colitis. **(A)** Chronic DSS-induced colitis was induced through oral administration of 3% DSS in distilled water for 5 days, followed by regular drinking water three times as shown in the schematic. Mitochondria were isolated from C2C12 cells transfected with p3XFLAG-CMV-10-CRIF1 or mock vector and mitochondria (10 μg in 200 μL saline) were intraperitoneally injected 1 day before and on days 2, 4, 6, 9, 12, and 14 from the start of treatment (n = 5/group). **(B)** Percentage change in body weight during the disease process. **(C)** On day 38 after the first DSS administration, colon sections were stained with hematoxylin and eosin (H&E) and antibodies against CRIF1, α-smooth muscle actin (α-SMA), and collagen-I (Col-I). Representative images are shown (original magnification: H&E 200×; IHC 400×, scale bar: 100 µm). Graphs show the mean histological scores for H&E and the numbers of antibody-positive cells for IHC. **(D)** On day 38 after the first DSS administration, the frequencies of IL-17+CD4+ and CD5+CD1d+IL-10+CD19+ cells in *ex vivo* spleens of mice were analyzed via flow cytometry. **(E)** CCD-18Co cells were transfected with pCMV6-Myc-DDK hCRIF1 vector or mock vector; 3 days later, mitochondria were isolated from these cells. Mitochondria (0.3 μg/mL) were cocultured with human PBMCs under the stimulation of anti-CD3 antibody (2 μg/mL); 3 days later, the supernatant was harvested and the amount of IL-17 was measured by ELISA. Values are presented as means ± SDs. *P < 0.05, **P < 0.01. Data are representative of two independent experiments.

As shown in **Figure 1**, the level of CRIF1 decreased along with mitochondrial abnormalities in mice with colitis. We found that injection of exogenous mitochondria into these mice alleviated weight loss and reduced the infiltration of inflammatory cells in colon tissue (data not shown). To determine whether CRIF1-enriched mitochondria more effectively reduce disease severity, we transfected these mitochondria into C2C12 myoblast cells, then isolated the resulting mitochondria and transplanted them into mice with chronic colitis (**Figure 6A**). Weight loss was alleviated in the treated group, which exhibited less lymphocyte infiltration into intestinal tissue and the mitigation of crypt loss and ulceration (**Figures 6A, B**). Injection of CRIF1-overexpressing mitochondria increased the levels of CRIF1+TOM20+ mitochondria in colon tissue (**Supplementary Figure S2**). Additionally, the treatment reduced the numbers of Col-I+ cells and α-SMA+ cells in the colonic mucosa (**Figure 6C**), as well as the frequency of splenic Th17 cells, germinal center B cells, and IL-10-producing regulatory B cells (**Figure 6D**). To investigate the effect of CRIF1-overexpressing mitochondria on human immune cells, 0.3 μg/mL mitochondria isolated from CCD-18Co cells, a human fibroblast cell line isolated from normal colon tissue, were cocultured with PBMCs under anti-CD3 antibody stimulation. Mitochondria isolated from CRIF1-overexpressing cells decreased the amount of IL-17 produced by PBMCs compared with control mitochondria (**Figure 6E**). These results demonstrate that CRIF1-overexpressing mitochondria alleviate colitis.

### 3.7 Patients with ulcerative colitis exhibit decreased levels of CRIF1 with dysfunctional mitochondria

**Figure 7:**
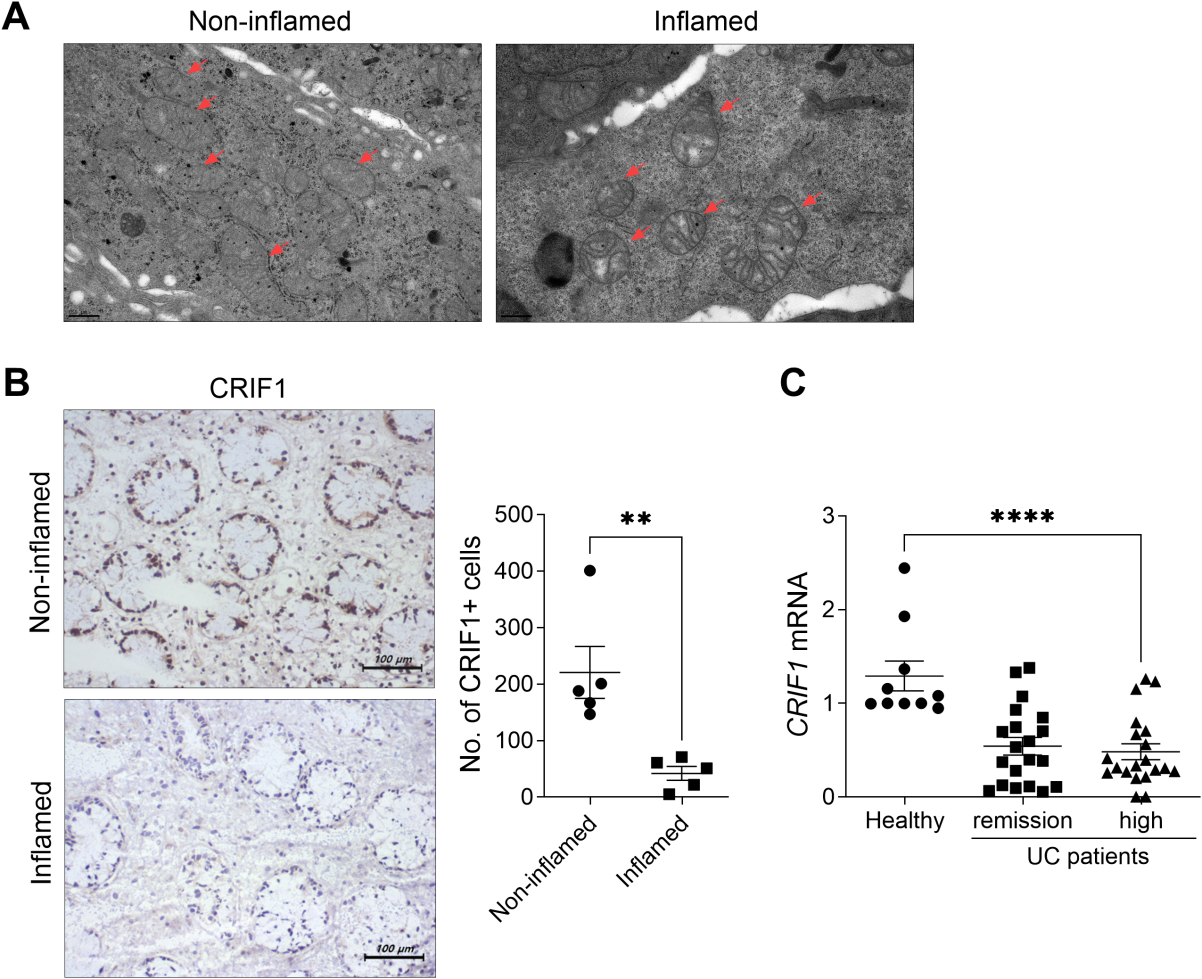
UC patients exhibit dysfunctional mitochondria and low CRIF1 mRNA levels. **(A)** Structural changes in mitochondria were examined via transmission electron microscopy in inflamed and non-inflamed colonic tissue sections of UC patients (left). **(B)** Colon tissue sections (n = 5/group) were subjected to immunohistochemistry using antibodies against CRIF1 (right). Representative images are shown. Graphs show the numbers of CRIF1-positive cells. **(C)** CRIF1 mRNA levels were examined by real-time polymerase chain reaction of PBMCs from healthy controls and UC patients with severe inflammation or remission. Values are presented as means ± SDs. **P < 0.01 and ****P < 0.0001. Data are representative of two independent experiments.

We observed morphological abnormalities such as swollen mitochondria with enlarged and broken cristae in the inflamed intestines of UC patients (**Figure 7A**). As observed in the intestinal tissue of DSS-induced colitis mice, levels of CRIF1 were significantly reduced in the inflamed intestine compared with the non-inflamed intestine of UC patients (**Figure 7B**). Additionally, PBMCs from UC patients with severe inflammation had a low level of CRIF1 mRNA relative to healthy controls (**Figure 7C**). These results suggest that mitochondrial CRIF1 plays a role in reducing inflammation and fibrosis in human immune cells and colonic fibroblast cells.

## 4 Discussion

We investigated a novel role for CRIF1 as a negative regulator of intestinal inflammation and fibrosis in IBD. In mice with DSS-induced colitis, CRIF1 levels in the intestine decreased as structural damage to mitochondria increased within the intestine during disease development. CRIF1 overexpression had a therapeutic effect, improving body weight and intestinal length loss and alleviating colonic inflammation, inflammatory cell death, and fibrosis. Additionally, transplantation of CRIF1-overexpressing mitochondria mitigated disease severity, and coculture of human-derived PBMCs with CRIF1-overexpressing mitochondria suppressed the frequency of pathogenic cells. Our findings indicate that mitochondrial dysfunction is involved in IBD development and reveal a previously unknown mechanism by which CRIF1 overexpression or transplantation of CRIF1-overexpressing, functional mitochondria may alleviate its progression.

CRIF1 is a nuclear and mitochondrial protein that regulates the cell cycle and growth and plays a role in the integration of mitochondrial OXPHOS polypeptides into the mitochondrial membrane (17, 18). Although no reports exist regarding the role of CRIF1 in intestinal inflammation, one report has addressed its role in intestinal development. CRIF1 is strongly expressed in the intestinal epithelium, where disruption of the CRIF1 gene causes abnormal differentiation of the small intestine and can lead to perinatal death (19). These results suggest that CRIF1 plays an essential role in intestinal development. Several reports indicate that CRIF1 plays a role in protecting against inflammatory responses. Myeloid-specific loss of CRIF1 causes an increase in M1-like macrophages that produce proinflammatory mediators and promote adipose inflammation and insulin resistance (26). In a previous study, we observed that CRIF1 deficiency in CD4+ cells increased the frequency of pathogenic Th17 cells by increasing STAT3 phosphorylation; Additionally, B cell-specific deletion of CRIF1 exacerbates lupus severity by promoting the production of IL-17 and IL-6, and CRIF1 overexpression suppresses lupus development in roquin^san/san^ mice, an experimental lupus model (27). In the present study, we demonstrated the therapeutic efficacy of CRIF1 in intestinal inflammation. CRIF1 gene therapy alleviated the decrease in body weight, loss of intestinal length, and fibrosis in mice with DSS-induced colitis. It also lowered the frequency of Th17 cells in peripheral blood and spleen and reduced the infiltration of IL-6+, IL-1β+, or VEGF+ cells in spleen tissue from these mice.

Intestinal epithelial cells require a tightly regulated mechanism between cell proliferation and death to protect their barrier function; abnormal cell death is an important factor causing IBD (28, 29). For example, excessive death of epithelial cells via necroptosis, a nonapoptotic programmed type of cell death linked to the activity of RIPK3 and MLKL, promotes intestinal inflammation (30). In a previous study, we found increased necroptosis in the colon of an experimental colitis mouse model, but this was alleviated by RIPK3 inhibition (25). In the present study, CRIF1 overexpression reduced the expression of factors related to necroptosis, such as RIPK3 and MLKL, in the intestine. In previous studies, CRIF1-deficient embryos showed defective cellular proliferation and increased cell death (17), and CRIF1 overexpression reduced amyloid-β-mediated cell death in neuroblastoma cell lines (31). Our results demonstrate that CRIF1 deficiency can promote necroptosis and suggest that this characteristic may contribute to IBD severity. However, there remains insufficient research concerning the relationships of CRIF1 with various types of cell death, including necroptosis; further studies are needed to determine the detailed molecular mechanisms by which CRIF1 participates in inflammatory cell death.

Mitochondria are central, dynamic organelles that maintain energy metabolism and cellular homeostasis by generating ATP through oxidative phosphorylation. They also play a role in cellular homeostasis by regulating calcium, apoptosis, ROS, and inflammatory signaling pathways (32, 33). Mitochondrial dysfunction is involved in the pathophysiology of IBD. Ultrastructural abnormalities are observed in the mitochondria of enterocytes and goblet cells of active CD patients (14). The activities of complexes II, III, and IV of the electron transport chain are reduced in the colonic mucosa of UC patients (34); the mitochondrial unfolded protein response, a mitochondrial stress pathway, is activated in intestinal epithelial cells of IBD patients (16). Mitochondrial impairment due to a deficiency of prohibitin 1, a mitochondrial chaperone protein required for optimal electron transport chain function, promotes the development of ileitis in conjunction with changes in the gut microbiota, and restoration of butyrate prevents ileitis (35). Additionally, NSAIDs induce mitochondrial stress and mitophagy in intestinal epithelial cells, leading to the release of mitochondrial DAMPs with pro-inflammatory potential (36); thus, the restoration of mitochondrial function is expected to be effective in IBD treatment. Recent research has focused on IBD alleviation by regulating mitochondrial function. Rosiglitazone, a PGC1A activator, has demonstrated efficacy for UC (NCT00065065) (37), and phase 2b clinical trials are underway in UC patients using MitoQ, an antioxidant derivative of coenzyme Q10 that inhibits mitochondrial ROS production (MARVEL study: NCT04276740) (38). In this study, we found that the expression of CRIF1 in intestinal tissue significantly decreased with increasing mitochondrial modifications during the development of DSS-induced colitis. CRIF1 was nearly undetectable in the splenic mitochondrial fraction from mice with colitis. Furthermore, patients with UC exhibit decreased levels of CRIF1 and dysfunctional mitochondria. We also found that injection of exogenous CRIF1-overexpressing mitochondria effectively mitigated disease in mice with DSS-induced colitis. Moreover, CRIF1-overexpressing mitochondria reduced the levels of inflammatory cytokines in human PBMCs *in vitro*. These results demonstrate that CRIF1 can alleviate the development of IBD by protecting the host from inflammation and dysfunctional mitochondria.

The DSS-induced colitis model used in this study is widely used in IBD research due to its rapidity, simplicity, reproducibility, and controllability (39). However, this model has a limitation in that, unlike human disease, T or B cell responses are not required for the development of the disease. Further studies in various disease models are needed to elucidate the complex role of CRIF1.

In summary, this is the first report that CRIF1 can alleviate the development of IBD by inhibiting inflammation, inflammatory cell death, and fibrosis, while improving mitochondrial function. Although the mechanism of action requires further exploration, the efficacy of CRIF1 or CRIF1-overexpressing mitochondria in controlling the development of IBD has been identified. Therefore, CRIF1 can serve as an effective treatment strategy for intestinal inflammatory disease, particularly IBD.

## Data Availability

The original contributions presented in the study are included in the article/**Supplementary Material**. Further inquiries can be directed to the corresponding authors.
