# Supplementary material for: CRIF1 gene therapy ameliorates inflammatory bowel disease by suppressing TH17 cells and fibrosis through mitochondrial function regulation
Source: Front Immunol. 2025 Jul 31;16:1618012. doi: 10.3389/fimmu.2025.1618012 (PMC12350146; doi:10.3389/fimmu.2025.1618012)
Supplement: Supplementary file 1 [file DataSheet1.pdf]

Supplementary table 1. Clinical characteristics of UC patients

Characteristics	No. (%)
No. of patients	23
Mean age, yr	41.7
Sex, F/M	9/14
Mean disease duration, yr	10.6
Severity	
Mayo Endoscopic Score	
Remission (0-1)	15 (65)
Mild (2-4)	8 (35)
Histologic score	
Chronic inflammatory infiltrate only	9 (39)
Mild activity (few neutrophils)	0 (0)
Moderate activity (neutrophils, epithelial damage)	5 (22)
Severe activity (erosions, ulceration)	9 (39)
Previous treatment	
Mesalazine	16 (70)
Steroids	7 (30)
Anti-TNF-α	4 (17)
Tofacitinib	2 (9)
Vedolizumab	1 (4)
