# Supplementary figures and images for: CRIF1 gene therapy ameliorates inflammatory bowel disease by suppressing TH17 cells and fibrosis through mitochondrial function regulation
Source: Front Immunol. 2025 Jul 31;16:1618012. doi: 10.3389/fimmu.2025.1618012 (PMC12350146; doi:10.3389/fimmu.2025.1618012)

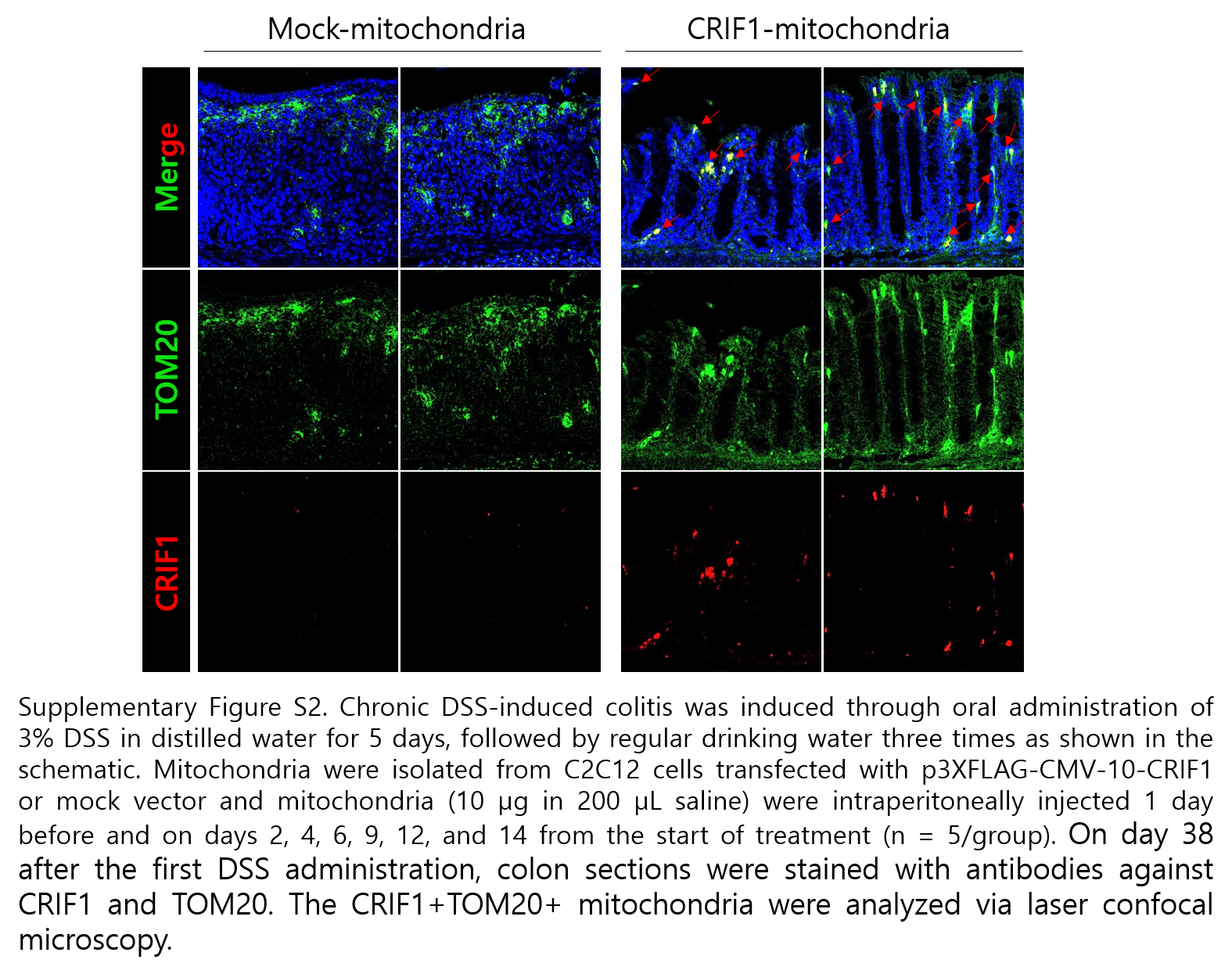

Supplement: Supplementary file 2 [file Presentation1.zip › Image 2.TIF]

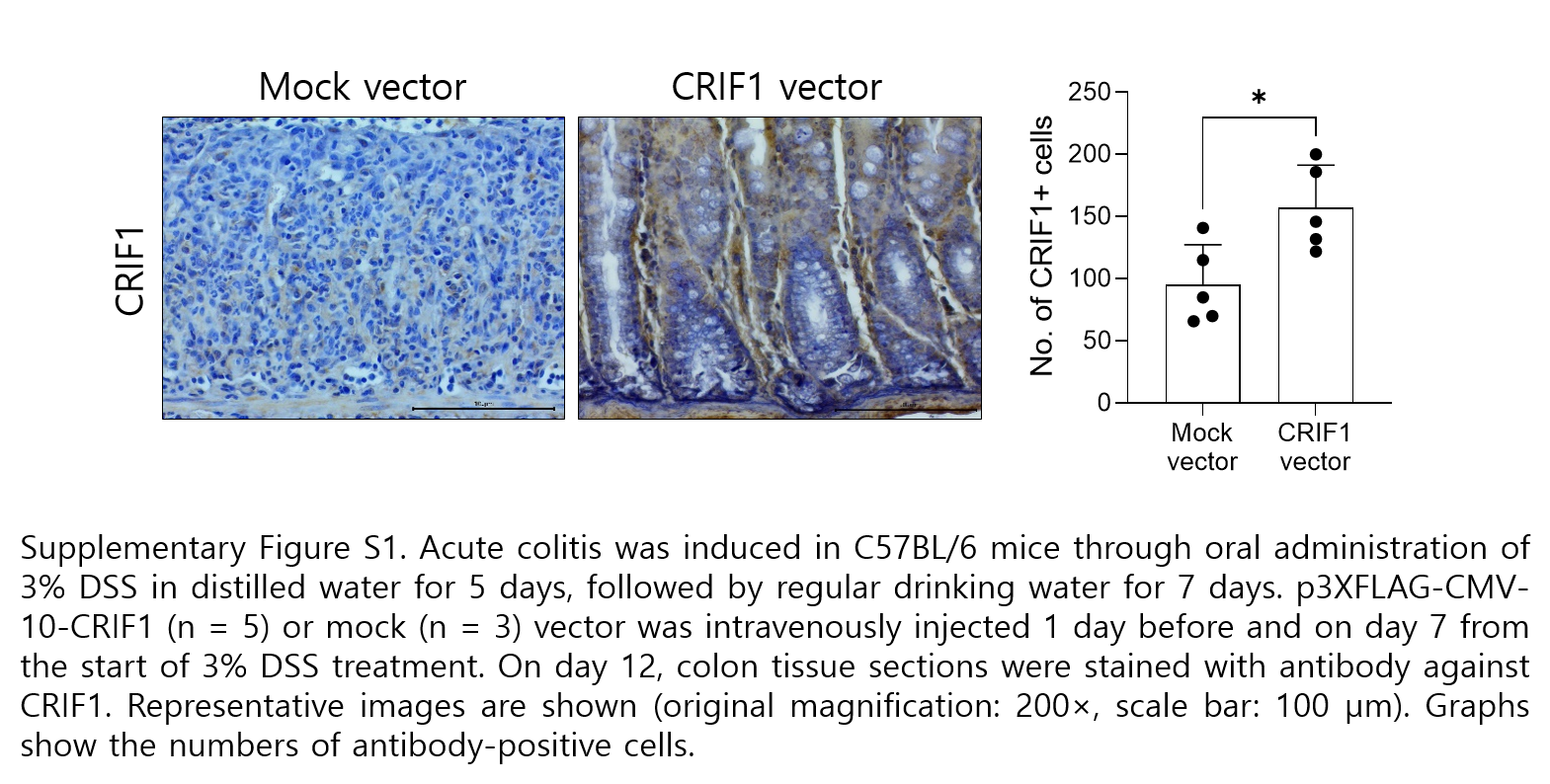

Supplement: Supplementary file 2 [file Presentation1.zip › Image 1.TIF]
